# One-pot synthesis of block-copolyrotaxanes through controlled *rotaxa*-polymerization

**DOI:** 10.3762/bjoc.13.127

**Published:** 2017-07-03

**Authors:** Jessica Hilschmann, Gerhard Wenz, Gergely Kali

**Affiliations:** 1 Organic Macromolecular Chemistry, Saarland University, Campus C4.2, 66123 Saarbrücken, Germany

**Keywords:** block copolymer, cyclodextrin, polyisoprene, polyrotaxane, RAFT polymerization

## Abstract

The aqueous reversible addition fragmentation chain-transfer (RAFT) copolymerization of isoprene and bulky comonomers, an acrylate and an acrylamide in the presence of methylated β-cyclodextrin was employed for the first time to synthesize block-copolyrotaxanes. RAFT polymerizations started from a symmetrical bifunctional trithiocarbonate and gave rise to triblock-copolymers where the outer polyacrylate/polyacrylamide blocks act as stoppers for the cyclodextrin rings threaded onto the inner polyisoprene block. Statistical copolyrotaxanes were synthesized by RAFT polymerization as well. RAFT polymerization conditions allow control of the composition as well as the sequence of the constituents of the polymer backbone which further effects the CD content and the aqueous solubility of the polyrotaxane.

## Introduction

Polymer necklaces, i.e., polyrotaxanes and pseudopolyrotaxanes, are supramolecular assemblies comprising polymeric axes with macrocycles threaded on them [1–4]. In the case of polyrotaxanes, the dethreading of the macrocycles is prevented by bulky stopper groups placed along the chain or at the chain ends. The importance of these supramolecules lies in the possibility to modify the properties, or even cross-link polymers without chemical modification of the backbone. Through polyrotaxane formation solubility [2–5], as well as mechanical [1–3,6–9] and electrical properties [10], can be improved. Cross-linking of threaded macrocycles gives rise to so-called slide-ring gels with unique mechanical properties [6,11–13]. One of the most important class of important macrocycles, applied in polyrotaxane chemistry, are cyclodextrins (CDs) because they are nontoxic, biodegradable and available in industrial scale. Furthermore, CDs can be simply functionalized by modification of the hydroxy groups [14].

There are several CD-based polyrotaxanes known with homo- and block-copolymer axes, mostly based on poly(ethylene oxide), poly(propylene oxide) or their copolymers [15–21], since these polymers can form sufficiently stable complexes with CDs. The application potential of these polyrotaxanes was already investigated in the fields of biomedicine, as drug [22] or gene [23] delivery vehicles, or in materials sciences, as slide ring gels [6]. Polyrotaxanes are mostly synthesized by a threading approach [2], a multistep method starting from pre-synthesized (co)polymers. Due to the hydrophobic interaction, as the driving force of complex formation, the threading of the CDs is only achievable in aqueous solution, but the hydrogen bonds between the hydroxy groups impede the water solubility of the products. Thus, the stoppering reaction is mostly limited to organic solutes, in which dethreading already takes place. This multistep reaction methodology hinders the large-scale production and broad application of these materials.

Recently our group has developed a method for a simple and environmentally friendly synthesis of polyrotaxanes. This, so called *rotaxa*-polymerization, is an aqueous, free radical copolymerization of a hydrophobic monomer, complexed in a host, with a stopper comonomer [24–25]. This latter has to be large enough to prevent the dissociation of the growing axis and the host, as it happens in the case of aqueous CD assisted homopolymerizations of hydrophobic monomers [26–28] including dienes [29]. This approach drastically widens the range of suitable hydrophobic polymeric axes, to all monomers being complexed in CD or hydrophilic CD derivatives. Up to now, *rotaxa*-polymerization was only performed via free radical reaction without control of the polymer chain length as well as statistical distribution of stopper groups along the axis. Herein, we report for the first time a simple one-pot synthesis of polyrotaxanes with control of length and sequence of the polymer axis through RAFT *rotaxa*-polymerization of isoprene in water. RAFT polymerization was indeed already started from a PEG α-CD pseudopolyrotaxane, but unthreading of α-CD was found to be a severe problem during polymerization, which could only be overcome by elaborate attachment of “molecular hooks” to both chain ends [30]. Furthermore, polyisoprene is advantageous for biomedical applications because of its high biocompatibility and biodegradability [31–33]. Here should be noted that polyisoprene was already subjected to pseudopolyrotaxane formation, with limited success, i.e., with β-CD only oligoisoprenes (degree of polymerization < 9) could form complexes with low coverage (3.0%) [34].

RAFT polymerization is a useful tool to form well-defined block-copolymers starting from a chain transfer agent (CTA) that drastically reduces the actual radical concentration in a fast equilibrium reaction [35–37]. This controlled polymerization should be advantageous for this work, compared to other polymerization techniques, such as atom transfer radical polymerization, because of the lack of toxic metal additives, and because of good control exerted in aqueous solution. The water-soluble bifunctional CTA *S*,*S*′-bis(α,α′-dimethyl-α′′-acetic acid)trithiocarbonate (DMATC) was selected because it allows synthesis of symmetrical triblock-copolymers in two steps. Other benefits of trithiocarbonate CTAs are the good hydrolytic stability [38], as well as their approved application in controlled isoprene polymerization [39–40]. Randomly methylated β-CD (RAMEB) was chosen as the CD derivative for this polyrotaxane synthesis since it is highly water-soluble and provides a sufficiently high binding constant for isoprene [24–25].

## Results and Discussion

**Scheme 1:**
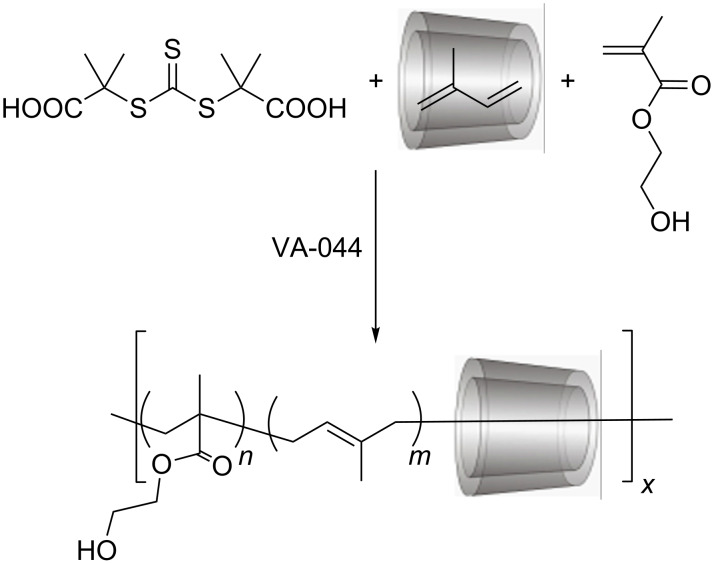
Synthesis route of RAMEB based statistical polyrotaxane.

In a first trial, statistical RAFT copolymerization of isoprene complexed in RAMEB was performed with water-soluble bulky comonomers, namely *N*-[tris(hydroxymethyl)methyl]acrylamide (TRIS-AAm) or 2-hydroxyethyl methacrylate (HEMA), shown in Scheme 1. The role of these bulky comonomers was to prevent the unthreading of RAMEB rings from the polymeric axis during and after polymerization. The resulting statistical copolymers were clearly soluble in water thus they could be purified by ultrafiltration. Besides water, the polyrotaxanes were also soluble in DMSO and less polar solvents, such as THF and chloroform. The weight fraction of RAMEB (Table 1) in the product was quantified from the optical rotation of a solution of the polymer (for the detailed description see Supporting Information File 1). The eventual content of free RAMEB in the product was checked by isothermal calorimetry (ITC) and was around 3 wt %. The conversion of the monomers was calculated from the yield of polyrotaxane minus the total RAMEB content. In both cases, the monomer conversions were around 60 wt % and the amount of threaded CD ranged between 47 and 65 wt %. These compositions were also supported by the integrals of the ^1^H NMR spectra (Supporting Information File 1).

**Table 1:** Yields, CD contents and weight average molar masses (*M_prx_*), degrees of polymerization (*P_w_*) and isoprene/stopper ratios (*i*/*st*) and dispersities (*Ð*) of the produced RAMEB based polyisoprene polyrotaxanes.

Polymer no.	comonomer	Yield (wt %)	RAMEB content (wt %)	*M_prx_*^a^ (kDa)	*P_w_*	*i*/*st*^b^	*Ð*

**1**	TRIS-AAm	67	47	40.5	270	8.5	1.8
**2**	HEMA	58	60	41.0	205	4.6	2.2

^a^Molar mass calculated from the molar mass of the acetylated polyrotaxanes measured by GPC; ^b^*i*/*st*: molar ratio of isoprene/stopper in the polymer as calculated from ^1^H NMR.

**Figure 1:**
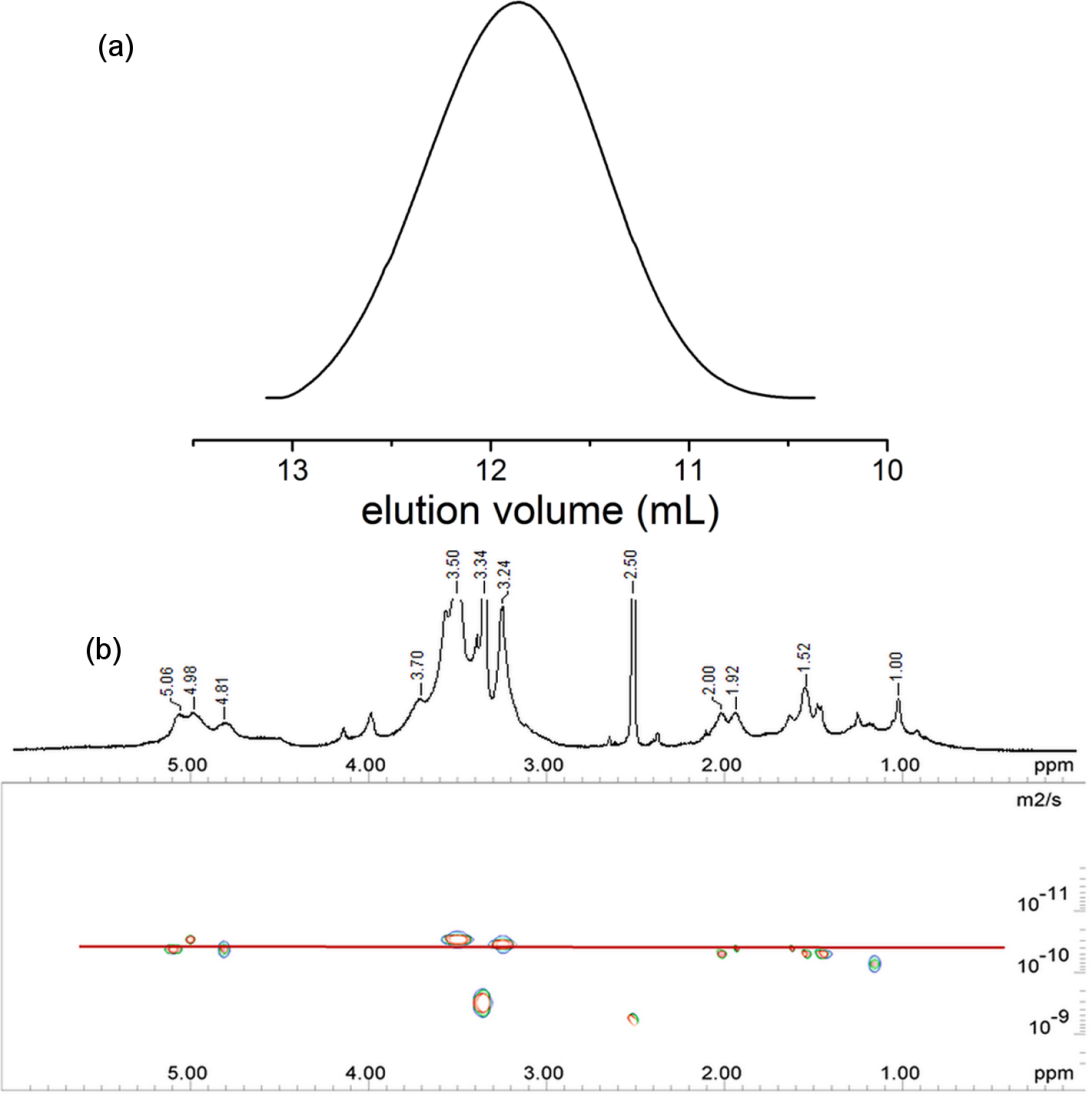
(a) GPC trace of the polyHEMA-*co*-polyisoprene polyrotaxane **1** and (b) 500 MHz ^1^H NMR and DOSY spectra of poly(TRIS-AAm)-*co*-polyisoprene polyrotaxane **2** in DMSO-*d*_6_.

The restricted mobility of the threaded macrocycles should lead to peak broadening in the ^1^H NMR spectra of the polyrotaxanes [41]. This peak broadening was indeed observed in the ^1^H NMR spectra of both polymers (Figure 1b and Supporting Information File 1) and was regarded as the first indication for a polyrotaxane structure. In addition, the agreement of the diffusion coefficients *D* for all proton NMR signals of the polymeric axis, RAMEB and the stopper in the diffusion-ordered NMR spectrum (DOSY, Figure 1b) further proved the existence of the polyrotaxane [42]. The *D* values of 1.0 × 10^−10^ and 4.1 × 10^−11^ m^2^/s were found for TRIS-AAm, and HEMA stoppered polyrotaxanes, respectively. The weight average molar mass *M_w_* determined by GPC was in both cases around 40 kDa, which means that the choice of the stopper was not critical for the composition and the size of the polyrotaxane. The amount of stopper integrated into the polyrotaxane was indeed difficult to quantify because of superposition of the ^1^H NMR signals with the ones of RAMEB, but the molar ratio isoprene/stopper (*i*/*st*) in the polymer could be estimated based on the integral values from the ^1^H NMR spectrum, using the region A (0.5–2.5 ppm) of the polymer backbone and the corresponding peaks of the stopper comonomers (Supporting Information File 1). Based on this calculation the estimated *i*/*st* values 8.5 and 4.6 were obtained for polyrotaxanes **1** and **2**, respectively. Also, the C=O vibrations of the polyacrylate units in the IR spectra of the polyrotaxanes were indicative for the integration of the stopper units into the polymer (see Supporting Information File 1).

The molar masses of polyrotaxanes **1** and **2** were determined after complete acetylation by GPC in THF (for results see Table 1). From the molar ratio *i/st*, the average molar mass per monomer repeat was derived 
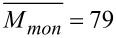
 Da for both polyrotaxanes **1** and **2**, which allows to calculate the degree of polymerization *P_w_* of the polymer backbone according to 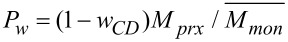 the values of *P_w_* were in reasonable agreement with the molar ratio of the related monomer vs CTA (175), which was indicative for a significant control of the radical polymerization by the CTA. The observed polydispersity indices *M_w_/M_n_* = 1.8–2.2 being higher than for regular RAFT polymerizations was attributed to a broad distribution of the number of methylated CD rings threaded on the polymer chains and the additional distribution of the number of methyl groups in the randomly methylated β-CD.

**Scheme 2:**
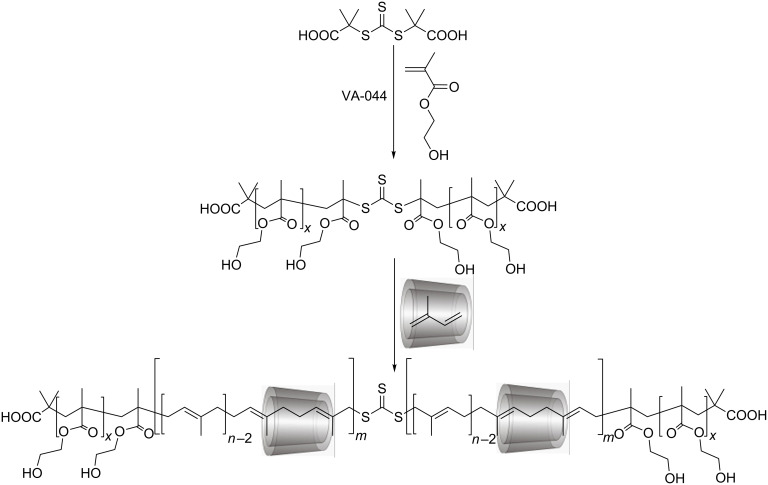
Schematic representation of the synthetic procedure for the preparation of randomly methylated β-CD based block-copolymeric polyrotaxane.

After the success of a *rotaxa*-RAFT polymerization, we investigated the synthesis of ABA triblock-copolyrotaxanes from the same building blocks in a two-step process as displayed in Scheme 2. First, one of the stopper monomers was homopolymerized by RAFT process starting from the bifunctional CTA, DMATC. In the second step, isoprene, complexed in RAMEB, was further polymerized utilizing the homopolymeric polyHEMA or polyTRIS-AAm as macro CTAs in water. Since the resulting block-copolyrotaxanes were nearly insoluble in water, they could be isolated through simple heat filtration, i.e., were heated up to 80 °C and filtered off at this temperature. Since the polymers were soluble in DMSO, the composition could be investigated by polarimetry and ^1^H NMR spectroscopy (see Table 2) as described in the first part.

**Table 2:** Yields, RAMEB content and weight average molar masses (*M_prx_*) and degrees of polymerization (*P_w_*) of the polymers obtained by RAFT polymerization.

Polymer no.	CTA	Monomer	Molar ratio monomer/CTA	Yield (wt %)	RAMEB (wt %)	*M_prx_*^a^ (kDa)	*Ð*	*P_w_* of new block^b^

**3**	DMATC	HEMA	16	95	0	9	2.5	21
**4**	**3**	isoprene	77	48	49	47	2.8	62
**5**	DMATC	TRIS-AAm	16	90	0	4	1.8	29
**6**	**5**	isoprene	80	51	65	45	1.9	77

^a^Molar mass calculated from the molar mass of the acetylated polyrotaxanes measured by GPC; ^b^*P_w_* of the new polyisoprene block was calculated from ^1^H NMR.

**Figure 2:**
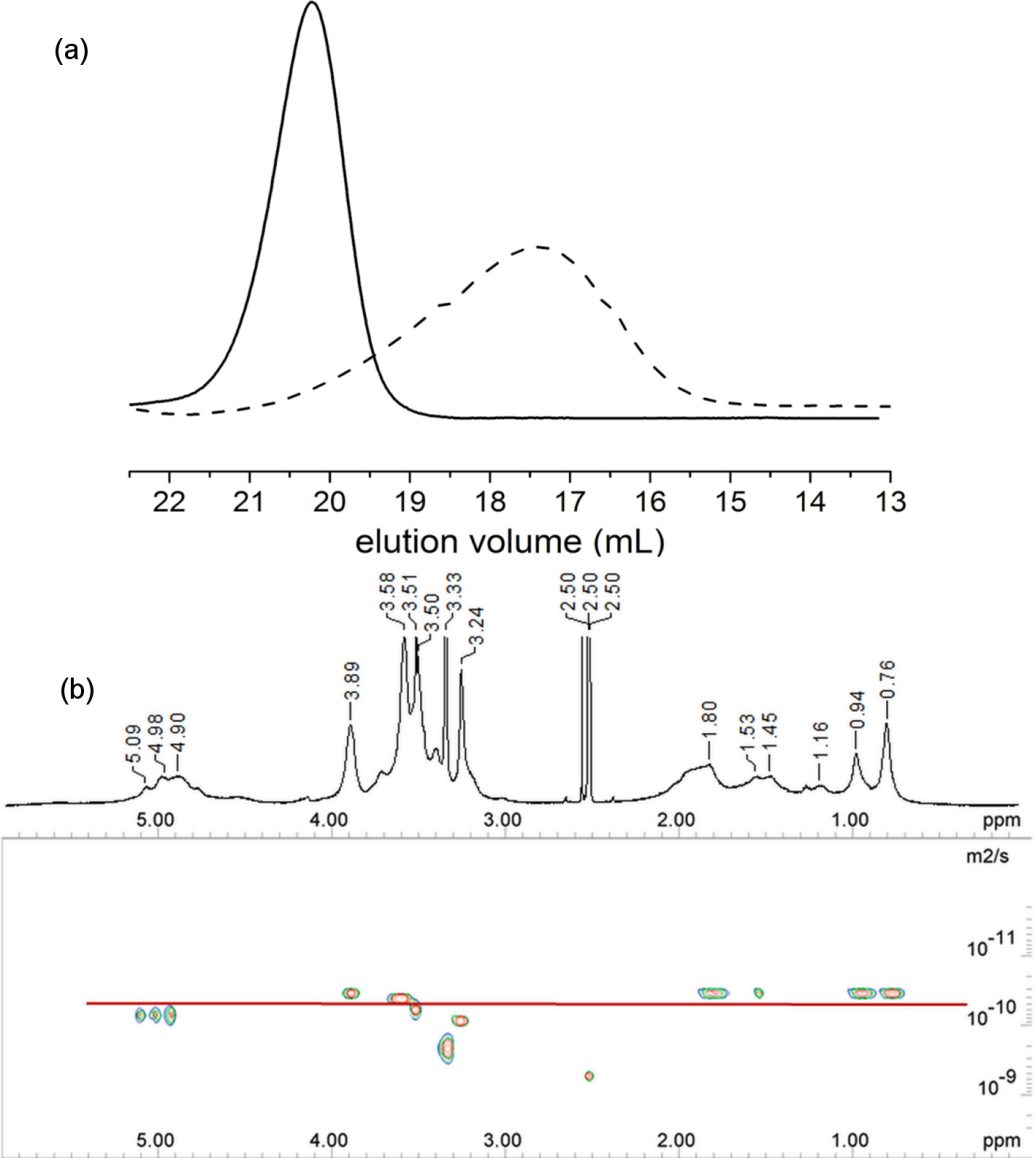
(a) GPC traces of the macroCTA **5** (solid line) and the poly(TRIS-AAm)-*b*-polyisoprene-*b*-poly(TRIS-AAm) polyrotaxane **6** (dashed line), and (b) 500 MHz, ^1^H NMR and DOSY spectra of polyHEMA-*b*-polyisoprene-*b*-polyHEMA polyrotaxane **4** in DMSO-*d*_6_.

The ^1^H NMR spectrum (Figure 2b) of the polymer **4** shows the signals of all RAMEB constituents at 3.0–4.1 and 4.5–5.0 ppm, polyisoprene at 1.0–2.3 and 5.0 ppm and of polyHEMA at 0.7–2.1 and 3.3–4.0 ppm. The noticeable peak broadening again is indicative of the formation of ABA triblock-copolyrotaxane. The DOSY measurements were carried out for the block-copolymer polyrotaxanes in DMSO. The same diffusion coefficients were detected for all components, such as RAMEB, isoprene, and stopper segments, verifying the polyrotaxane formation, as presented in Figure 2b. Diffusion coefficients of both block-polyrotaxanes were around 3.0 × 10^−11^ m^2^/s, due to the similar molar masses for both polyrotaxanes after block-copolymerization.

The molar masses of polyrotaxanes **3**–**6** were determined after complete acetylation by GPC in THF (Table 2). First, after homopolymerization, the molar masses were 9 and 4 kDa for the polyHEMA and polyTRIS-AAm stoppers blocks, respectively. After block copolymerization with isoprene complexed in RAMEB, the corresponding molar masses increased significantly to 50 and 45 kDa, indicating further polymerization and coinciding polyrotaxane formation. Starting from both polyHEMA and polyTRIS-AAm CTAs, monomer conversions were around 50%. For these block-copolymers with polyrotaxane middle blocks, the CD contents were 49 and 65 wt % of RAMEB. For the polyHEMA based polyrotaxane **4**, this coverage was a bit lower than for the statistical copolymerization and also the obtained polyisoprene block length *P_w_* was slightly lower than the theoretical value. These deviations are most likely explained by the limited aqueous solubility and the high molar mass (9 kDa) of the polyHEMA CTA **3**. In contrast to HEMA-based block-copolyrotaxane, the coverage of polyTRIS-AAm-based block-copolyrotaxane **6** was higher than that of for the statistical one, while the molar mass remained the same, and the polyisoprene block length *P_w_* being close to the theoretical value (77 instead of 80). This indicates a good control of the CTA over the polymerization. The composition of the backbone *i/st* can be easily calculated from the molar masses of the blocks to the polymeric axes. These *i*/*st* ratios were 4.70 and 8.75 for polyrotaxanes **4** and **6**, respectively. The corresponding *i/st* values, estimated from the ^1^H NMR spectra were 3.00 and 8.30. The too low value obtained for the HEMA stoppered polyrotaxane was attributed to the superposition of the signals of –C*H*_2_-OH from HEMA and of RAMEB which hinders the accurate determination of the polymer composition. The polydispersity indices were again slightly higher than that of for regular RAFT polymerizations, 2.8 and 1.9 for polyrotaxanes **4** and **6**, respectively. This increased PDI is connected to the statistical threading of the cyclodextrin rings, which also have some mass distribution. The higher PDI 2.8 of the polyHEMA block-copolyrotaxane **4** was attributed to the low aqueous solubility of the polyHEMA CTA.

## Conclusion

The above-described procedure is the first controlled *rotaxa*-polymerization resulting in well-defined statistical and block-copolymeric polyrotaxanes. The method is versatile and should work for many monomers and CD derivatives. The resulting triblock-copolyrotaxanes might be good stocks for the synthesis of highly elastic slide-ring gels and hydrogels. Due to the biocompatibility of the constituents and the ability of the polyrotaxanes to self-assemble, these materials might also be applicable for drug delivery or tissue engineering.

## Supporting Information

File 1 General methods, experimental procedures, and characterization of compounds **1**–**6**.
