# Supplementary material for: One-pot synthesis of block-copolyrotaxanes through controlled rotaxa-polymerization
Source: Beilstein J Org Chem. 2017 Jul 3;13:1310–5. doi: 10.3762/bjoc.13.127 (PMC5530718; doi:10.3762/bjoc.13.127)
Supplement: File 1 — General methods, experimental procedures, and characterization of compounds 1–6. [file Beilstein_J_Org_Chem-13-1310-s001.pdf]

Supporting Information

for

One-pot synthesis of block-copolyrotaxanes through controlled *rotaxa*-polymerization

Jessica Hilschmann, Gerhard Wenz and Gergely Kali*

Address: Organic Macromolecular Chemistry, Saarland University, Campus C4.2, 66123 Saarbrücken, Germany

E-mail: Gergely Kali - gergely.kali@uni-saarland.de

*Corresponding author

General methods, experimental procedures, and characterization of compounds 1–6

1. Materials and methods S1
2. Syntheses and characterizations S5
   Polyrotaxane 1 .... S6
   Polyrotaxane 2 .... S9
   Polymer 3 and polyrotaxane 4 .... S12
   Polymer 5 and polyrotaxane 6 .... S14
3. Determination of the molecular weight distributions S17

1. Materials and methods

The monomers isoprene (99%, distilled), *N*-[tris(hydroxymethyl)methyl]acrylamide (93%), 2-hydroxyethyl methacrylate (97%, distilled) as well as tetrabutylammonium hydrogen sulfate (97%) were purchased from Sigma-Aldrich. Randomly methylated β-CD (RAMEB, 99%, degree of methylation 1.6 per glucose unit, dried under vacuum at 65 °C) was donated by Wacker. 2,2'-Azobis[2''-(2'''-imidazolin-2'''-yl)propane] dihydrochloride (VA-044, 95%) initiator was obtained from Fluorochem, while carbon

disulfide (99.9%) was purchased from Acros Organics, and both were used without purification

^{1}H NMR measurements were performed on a *Bruker Advance Ultrashield 400* (400.2 MHz) instrument. The protons of RAMEB were denoted as H-1, H-2, …H-6 and O-CH_3, the protons of polyisoprene H-a, H-b….H-e, and the protons of stopper comonomers were denoted as H-A, H-B…H-D as shown in Figure S1.

Assignment of the protons of the polyrotaxanes for NMR

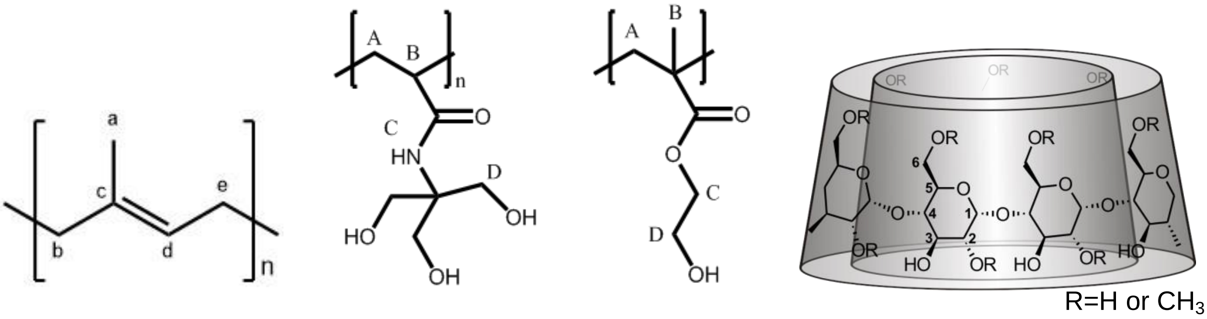

Scheme S1: Assignment for polyisoprene, poly(*N*-tris(hydroxymethyl)methyl]acrylamide), poly(2-hydroxyethyl methacrylate), and RAMEB.

DOSY was performed on a *Bruker AVANCE 500* at 296 K. The experiments were performed without spinning to avoid convection. The standard Bruker pulse program was ledbpgp2s, and the gradient recovery delay was held constant at 0.2 ms. The D20 value (diffusion time) was adjusted between 400 and 700 ms. The bipolar diffusion gradients (P30) were set between 1.5–3.0 ms and the number of gradient steps was set to 32. The data were first processed with *Topspin 3.2* software to obtain a phased and baseline-corrected spectra. Then, the diffusion dimension was generated with *Bruker Dynamics Center 2.2.4* via inverse Laplace transform (ILT).

Gel permeation chromatography (GPC) was used for the determination of the molecular weights and the molecular weight distributions of the polymers after acetylation of the OH groups at RAMEB and also the stopper comonomer. The GPC

system was equipped with an SDV 10^5 Å column from Polymer Standards Service, Mainz, Germany (PSS) and a Waters 2410 refractive index detector. For polyrotaxanes **1**, **6** and polymer **5** the GPC setup consisted of SDV 10^3 and 10^5 Å columns also from PSS. The mobile phase was THF, and the flow rate was 1.0 mL/min at 25 °C using a Viscotek VE1121 GPC pump. The column was calibrated with narrow linear polyisoprene standards (from 1,070 to 10^6 g mol^{-1}) from PSS. The calibration curves for the two systems are presented in Figure S2 (a) and (b). Calculations were performed with the program WinGPC Unity (PSS). The molar mass of the polyrotaxanes (M_{prx}) was calculated from the measured molar mass of the acetylated polyrotaxanes (M'_{prx}) from the equation:

$$M_{prx} = M'_{prx}\,(1 - w_{ac})$$

where the weight fraction of the acetyl groups (w_{ac}) was calculated according to the following equation from the molar masses of CD derivative and acetate, M_{CD} and M_{ac}, assuming full acetylation (8.75 OH groups of RAMEB, the degree of acetylation per CD, d_{ac} = 8.75).

$$w_{ac} = \frac{n_{CD} d_{ac} M_{ac}}{n_p M_p + n_{CD}(M_{CD} + d_{ac} M_{ac})} = \frac{w'_{CD} d_{ac} M_{ac}}{M_{CD} + w'_{CD} d_{ac} M_{ac}}$$

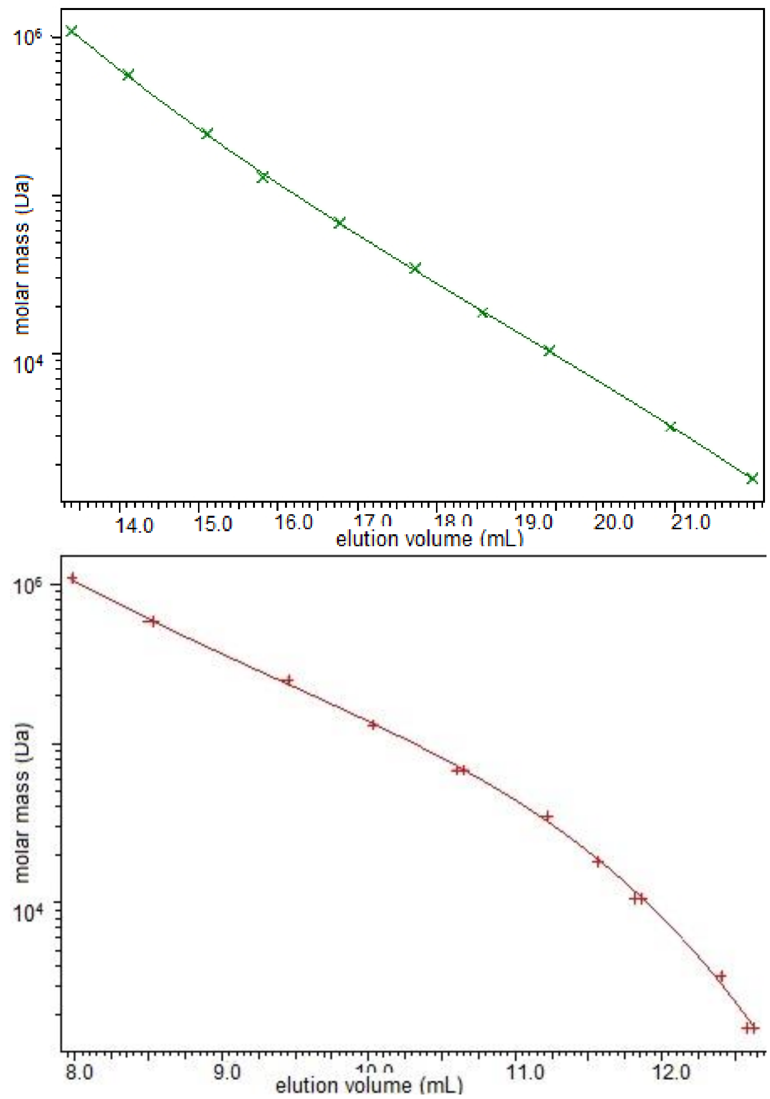

Figure S1: The calibrations curves used for the molar mass, and dispersity determination of the polymers and polyrotaxanes in this work for (a) SDV 10^3 Å plus SDV 10^5 Å column and (b) for SDV 10^5 Å column, both systems from PSS.

The **IR spectroscopy** measurements were performed using an FTIR spectrometer *Tensor 27* from *Bruker Optic GmbH* (Ettlingen, Germany) on samples cast from $CHCl_3$ solution onto a Ge chip. The FT data were treated by the *OPUS* program from the same company.

Optical rotation measurements were determined with a *PerkinElmer 241* polarimeter at λ = 589 nm at 25 °C in chloroform solution. For quantification of RAMEB, a calibration line with pure RAMEB in chloroform was determined. A specific rotation of $[\alpha]_{589}$ = 130 mLdm^{-1}g^{-1} was found for RAMEB.

Isothermal titration calorimetry (ITC) measurements were performed with the *Nano ITC^{2G}* device equipped with a gold cell at 25 ± 0.0002 °C from *TA Instruments*, New Castle, USA. The evolved heats were corrected by the corresponding heats of dilution and fitted by the program *NanoAnalyze* from *TA Instruments* using the algorithm for interactions with 1:1 stoichiometry. K_a was fixed at 21.3 × 10^3 M^{-1} for RAMEB, as described previously.[1]

The weight fraction of **threaded cyclodextrin** (w'_{CD}) was calculated as the difference of the total RAMEB content (w_{CD}), from the optical rotation measurements, and the amount of free cyclodextrin (w''_{CD}), determined by ITC, using adamantane-1-carboxylate guest molecule [1]

$$w'_{CD} = w_{CD} - w''_{CD}$$

Ultrafiltration was carried out using polyethersulfone cross flow membrane (cut-off molecular weight 10 kDa, Sartorius (Göttingen, Germany), *Vivaflow 200*) with EtOH/water 5:95 v/v.

2. Syntheses and Characterizations

S,S'-Bis(α,α'-dimethyl-α''-acetic acid)-trithiocarbonate

The symmetrical bifunctional CTA was prepared according to the literature [2]. In detail, 50% aqueous sodium hydroxide (201.6 g, 2.52 mol) was added dropwise to a mixture of carbon disulfide (27.4 g, 0.36 mol), chloroform (107.5 g, 0.90 mol), acetone (52.30 g, 0.90 mol), tetrabutylammonium hydrogen sulfate (2.41 g, 7.1 mmol) and 120 mL of *n*-hexane in a 500 mL three-neck round-bottom flask, under N_2. The mixture was stirred for 16 h, then 900 mL water and 120 mL *cc.* HCl was added. The dark-yellow precipitate was filtered and washed with distilled water. After recrystallization from toluene/acetone (4:1) mixture, 40.13 g (40%) light yellowish

solid was collected and identified as *S*,*S*'-bis(α,α'-dimethyl-α''-acetic acid)-trithiocarbonate) CTA.

^{1}H NMR (DMSO-d6): 1.59 (s, 12H), 12.91 (s, 2H)

Polyrotaxane ***1***

The initiator VA-044 (0.92 mg, 0.003 mmol), *S*,*S*'-bis(α,α'-dimethyl-α''-acetic acid)-trithiocarbonate) CTA (8.10 mg, 0.03 mmol), RAMEB (5.88 g, 4.5 mmol), *N*-[tris(hydroxymethyl)methyl]acrylamide (78.7 mg, 0.45 mmol) were dissolved in water (10 mL) under stirring in a vial equipped with Teflon covered septum. After bubbling N_2 through the solution for 20 min, isoprene (0.45 mL, 4.5 mmol) was added by a syringe into the vial, and the mixture was stirred until clear solution. The polymerization was initiated by heating to 40 °C and stirred for three days at this temperature. 530 mg of the white solid product was obtained after ultrafiltration and lyophilization.

IR = 3200-3600 cm^{-1} (O-H), 2800-3000 cm^{-1} (C-H) 1600-1700 cm^{-1} (C=O)

^{1}H NMR (DMSO-d_6, 400 MHz) δ/ppm = 7.40–6.70 (br m, low intensity H-C), 5.15–4.97 (H-d), 4.95–4.50 (m, 1.2H, H-1, OHs), 4.00–3.30 (m, 6H, H-2–H-6, H-D), 3.25 (s, 1.8H, O-CH_3), 2.05–1.85 (H-b, e) and 1.68–1.00 (H-a, A, B)

The total weight fraction w_{CD}= 50.2 wt % of RAMEB was determined from the optical rotation α = 0.073 deg of a solution (10.75 mg mL^{-1}) in DMSO at λ = 589 nm applying the measured specific rotation of Me-β-CD $[\alpha]_{589}$ = +130 mL dm^{-1}g^{-1}. For the determination of the content of free RAMEB (w''_{CD} = 3 wt %) a solution (total concentration of RAMEB 1.0 mM) of the polyrotaxane **1** in 0.1 M phosphate buffer was titrated at 25.000 °C with an 8 mM solution of the guest adamantane-1-carboxylate sodium salt monitored by ITC. The weight fraction of **threaded**

cyclodextrin (w'_{CD} = 47 wt %) was calculated as the difference of w_{CD}= 50.2 wt.% and the w''_{CD}= 3 wt.% [1].

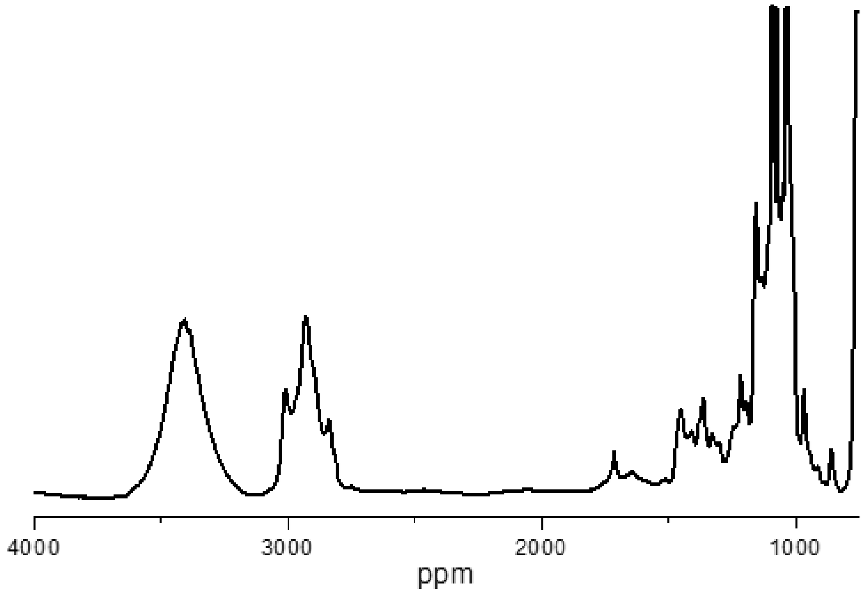

Figure S2: IR spectrum of the polyrotaxane **1**.

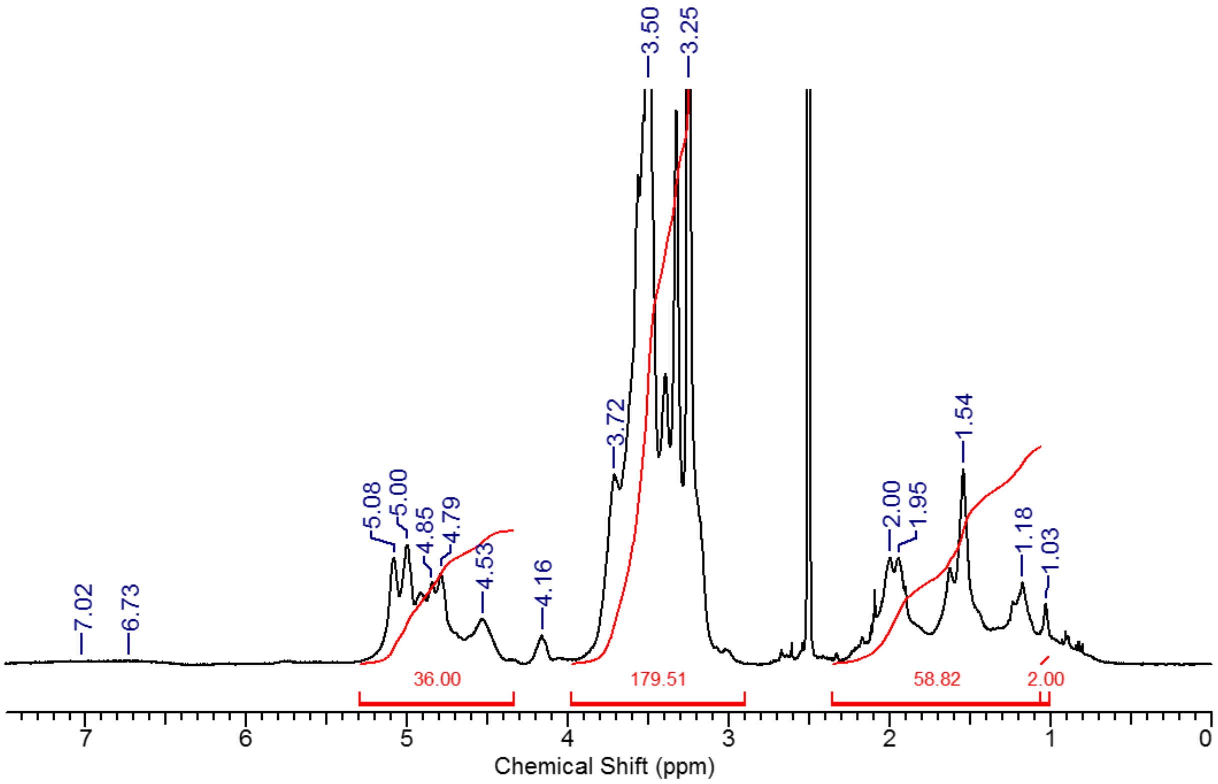

Figure S3: 400 MHz ^{1}H NMR of the polyrotaxane **1** in DMSO-d_6.

The isoprene/stopper molar ratio *i/st* = 8.5 = (58.82-1)/7 was calculated from the integral (normalized to the methylene signal at 1.03 ppm of TRIS-AAm backbone (H-A 2.00) of the residual signals of the polymeric backbone in region A (0.5–2.3 ppm) 58.82 containing 7 protons of the 1,4-isoprene repeat and 1 methine proton (H-B) from acrylamide repeating unit.

The integrals of regions of A (0.5–2.3 ppm), B (2.9–3.9 ppm) and C (4.75–5.25 ppm) were calculated according to the following equations.

$$\mathrm{A} = 60.82 = 7\frac{i}{CD} + 3\frac{stopper}{CD}$$

$$\mathrm{B} = 179.51 = 76 + 9\frac{stopper}{CD}$$

$$\mathrm{C} = 36.00 = \frac{i}{CD} + 9$$

The equations emerged from the following assignments: signals in region A are due to the 3 methyl- plus 4 methylene protons of the 1,4-isoprene repeat and 2 methylene plus 1 methine (H-A, B) from acrylamide (stopper) repeating unit. Signals in region B are from around 34 methyl protons (O-CH_3), 14 methylene plus 28 methine protons (H-2–6) of RAMEB plus 6 and 3 protons of the hydroxymethyl groups from the acrylamide-stopper (H-D, OH). Signals in region C are due to the 7 anomeric methine protons (H-1) and around 2 OH groups of RAMEB plus 1 methine proton (H-d) at the double bond of the 1,4-isoprene repeat. The ratios of the integrals of regions A, B, and C agreed well with the calculated ones.

Polyrotaxane ***2***

The initiator VA-044 (0.92 mg, 0.003 mmol), *S*,*S*'-bis(α,α'-dimethyl-α''-acetic acid)-trithiocarbonate) CTA (8.10 mg, 0.03 mmol), RAMEB (5.88 g, 4.5 mmol), 2-hydroxyethyl methacrylate (54.5 μL, 0.45 mmol) were dissolved in water (10 mL) under stirring in a vial equipped with Teflon covered septum. After bubbling N_2 through the solution for 20 min, isoprene (0.45 mL, 4.5 mmol) was added by a syringe into the vial, and the mixture was stirred until clear solution. The polymerization was initiated by heating to 40 °C and stirred for three days at this temperature. 530 mg of the white solid product was obtained after ultrafiltration and lyophilization.

IR = 3200-3600 cm^{-1} (O-H), 2800-3000 cm^{-1} (C-H) 1700-1750 cm^{-1} (C=O)

^{1}H-NMR (DMSO-d_6, 400 MHz) δ/ppm = 5.15–4.97 (H-d), 4.95–4.70 (s, 1.2H, H-1, OH), 3.98 (s, H-D), 3.85–3.30 (m, 6H, H-2–H-6, H-C), 3.25 (s, 1.8H, O-CH_3), 2.05–1.20 (H-a, b, e, A) and 1.00–0.88 (H-B)

The total weight fraction w_{CD}= 63.0 wt % of RAMEB was determined from the optical rotation α = 0.055 deg of a solution (6.50 mg mL^{-1}) in DMSO at λ = 589 nm applying the measured specific rotation of Me-β-CD $[\alpha]_{589}$ = +130 mL $dm^{-1}g^{-1}$. For the determination of the content of free RAMEB (w''_{CD} = 3 wt %) a solution (total concentration of RAMEB 1.0 mM) of the polyrotaxane **2** in 0.1 M phosphate buffer was titrated at 25.000 °C with an 8 mM solution of the guest adamantane-1-carboxylate sodium salt monitored by ITC. The weight fraction of **threaded cyclodextrin** (w'_{CD} = 60 wt %) was calculated as the difference of w_{CD} = 63.0 wt % and the w''_{CD} = 3 wt % [1].

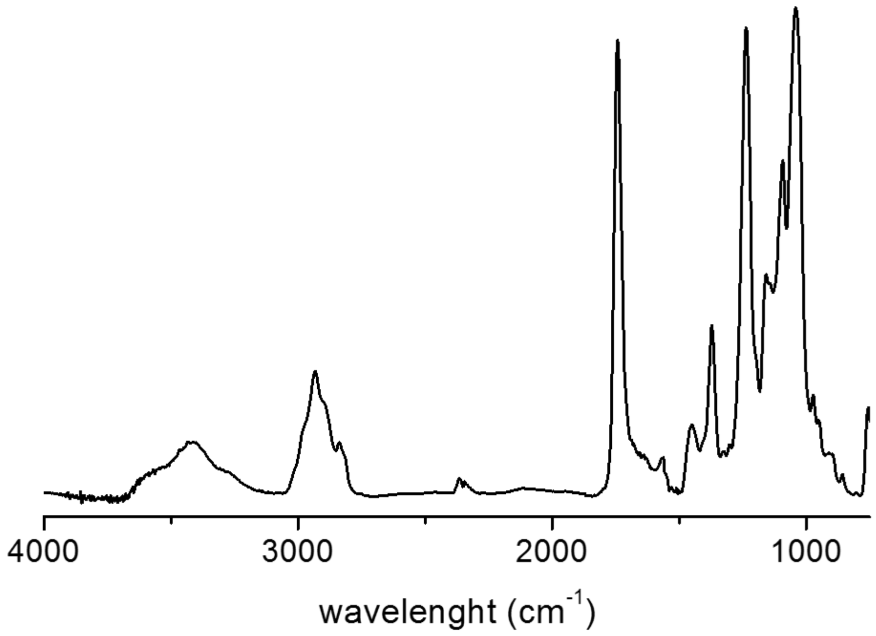

Figure S4: IR spectrum of the polyrotaxane **2**.

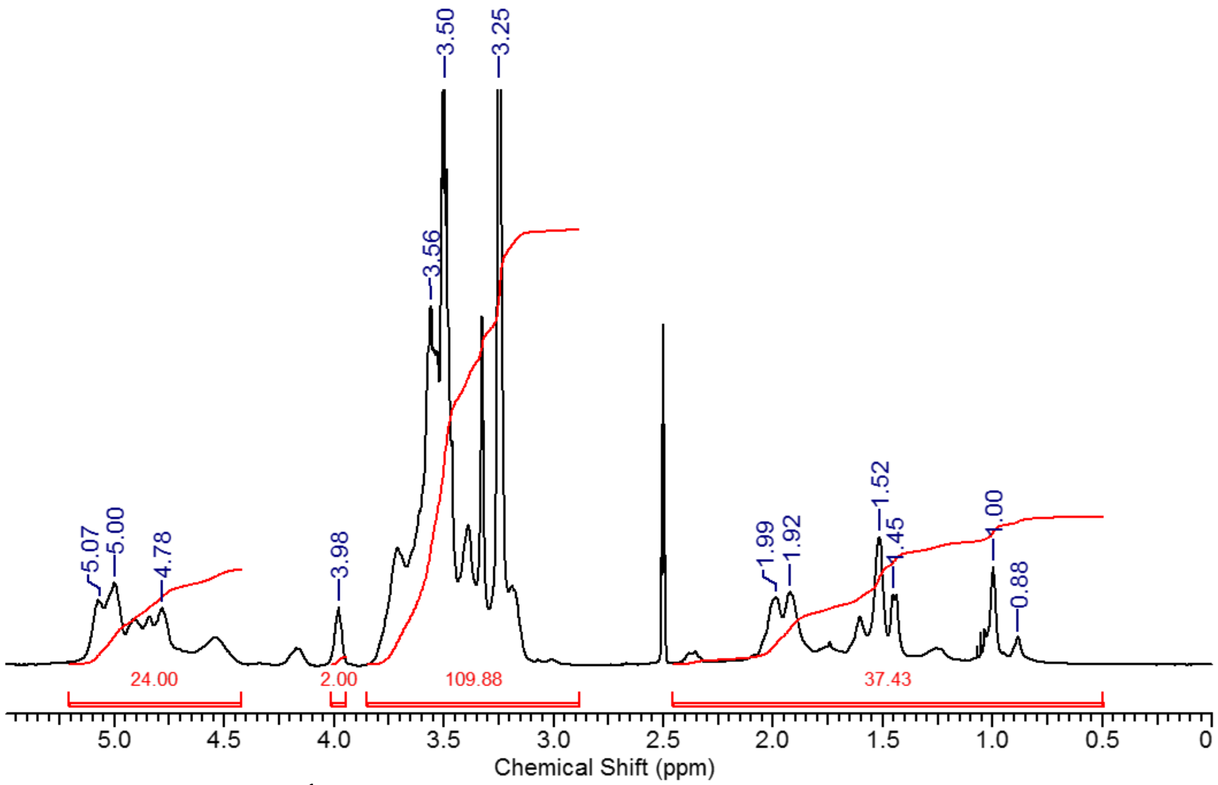

Figure S5: 400 MHz ^{1}H NMR of the polyrotaxane **2** in DMSO-d_6.

The isoprene/stopper molar ratio *i*/*st* = 4.6 = (37.43-5)/7 was calculated from the integral (normalized to the –CH_2OH methylene signal at 3.98 ppm of HEMA (H-D) 2.00) of the residual signals of the polymeric backbone in region A (0.5–2.3 ppm) 37.43 containing 7 protons of the 1,4-isoprene repeat and 5 protons (H-A,B) from methacrylate repeating unit.

The integrals of regions of A (0.5–2.3 ppm), B (2.9–3.9 ppm) and C (4.75–5.25 ppm) were calculated according to the following equations.

$$\mathrm{A} = 37.43 = 7\frac{i}{CD} + 5\frac{stopper}{CD}$$

$$\mathrm{B} = 109.88 = 76 + 2\frac{stopper}{CD}$$

$$\mathrm{C} = 24.00 = \frac{i}{CD} + 9$$

The equations as already described above, emerged from the following assignments: signals in region A are due to 7 protons of the 1,4-isoprene repeat and 2 methylene plus 3 methyl (H-A, B) from the backbone of HEMA repeating unit. Signals in region B are from around 76 protons of RAMEB plus 2 methylene protons of the HEMA-stopper (H-C). Signals in region C are due to 9 protons of RAMEB plus 1 methine proton of the 1,4-isoprene repeat. The ratios of the integrals of regions A, B, and C in this case also agreed well with the calculated ones.

*Polymer **3** and Polyrotaxane **4***

The initiator VA-044 (1.85 mg, 0.006 mmol), S,S'-bis(α,α'-dimethyl-α''-acetic acid)-trithiocarbonate) CTA (16.20 mg, 0.06 mmol), and 2-hydroxyethyl methacrylate (109 mL, 0.90 mmol) were dissolved in water (10 mL) under stirring in a vial equipped with septum. The oxygen was removed by bubbling N_2 through the solution. The polymerization was initiated by heating to 40 °C and stirred for three days at this temperature. The poly(2-hydroxyethyl methacrylate) was precipitated with cold ether and dried in a vacuo until constant weight. 95 mg of the white/light yellowish solid product **3** was obtained.

80 mg of polymer **3** macroCTA, 0.70 mg VA-044 (0.002 mmol), and 4.50 g RAMEB (3.4 mmol) were dissolved in water and bubbled trough N_2 in a vial equipped with Teflon covered septum. Isoprene (0.32 mL, 3.2 mmol) was added by a syringe into the vial, and the mixture was stirred until clear solution. The polymerization was initiated by heating to 40 °C and stirred for three days at this temperature. The resulting milky emulsion was heated for 20 mins at 80 °C; the white precipitate was isolated by filtration and lyophilized to obtain 260 mg of solid product.

IR = 3200-3600 cm^{-1} (O-H), 2800-3000 cm^{-1} (C-H) 1700–1750 cm^{-1} (C=O)

^1H NMR (DMSO-d_6, 400 MHz) δ/ppm = 5.15–4.97 (H-d), 4.95–4.70 (s, 1.2H, H-1, OH), 3.98 (s, H-D), 3.85–3.30 (m, 6H, H-2–H-6, H-C), 3.25 (s, 1.8H, O-CH_3), 2.05–1.20 (H-a, b, e, A) and 1.00–0.88 (H-B)

The total weight fraction w_{CD} = 54.5 wt % of RAMEB was determined from the optical rotation α = 0.061 deg of a solution (9.60 mg mL^{-1}) in $CHCl_3$ at λ = 589 nm applying the measured specific rotation of Me-β-CD $[\alpha]_{589}$ = +130 mL $dm^{-1}g^{-1}$. For the determination of the content of free RAMEB (w''_{CD} = 6 wt %) a solution (total concentration of RAMEB 1.0 mM) of the polyrotaxane **4** in 0.1 M phosphate buffer was titrated at 25.000 °C with an 8 mM solution of the guest adamantane-1-carboxylate sodium salt monitored by ITC. The weight fraction of **threaded cyclodextrin** (w'_{CD} = 49 wt %) was calculated as the difference of w_{CD} = 54.5 wt % and the w''_{CD} = 6 wt % [1].

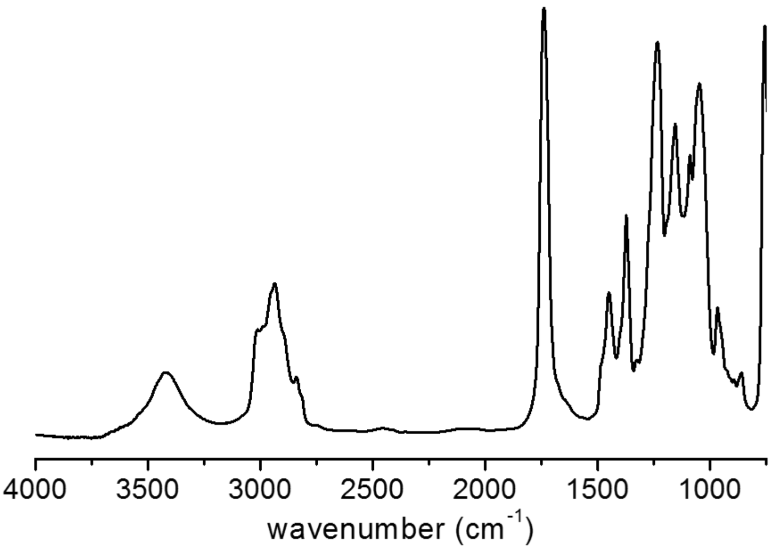

Figure S6: IR spectrum of the polyrotaxane **4**.

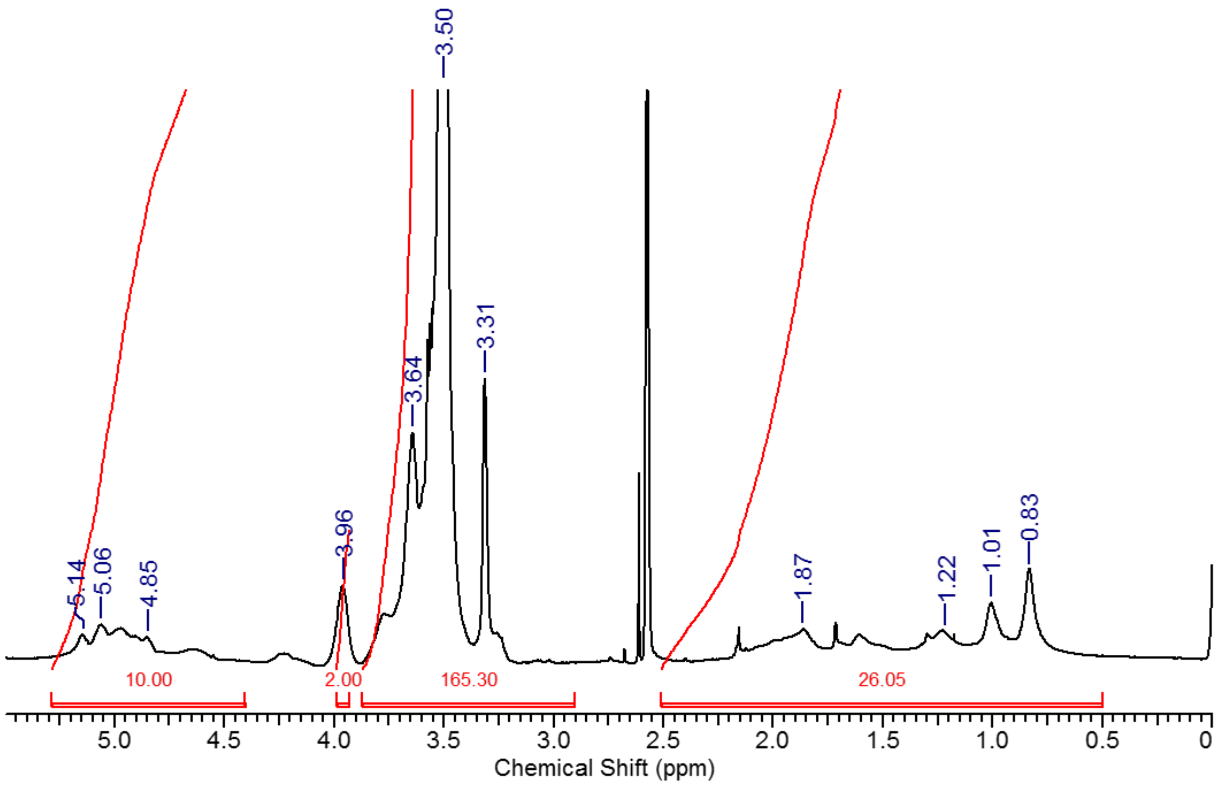

Figure S7: 400 MHz ^{1}H NMR of the polyrotaxane **4** in DMSO-d_6.

*Polymer **5** and polyrotaxane **6***

The initiator VA-044 (1.85 mg, 0.006 mmol), *S*,*S*'-bis(α,α'-dimethyl-α''-acetic acid)-trithiocarbonate) CTA (16.20 mg, 0.06 mmol), and *N*-[tris(hydroxymethyl)methyl]acrylamide (157.5 mg, 0.90 mmol) were dissolved in water (10 mL) under stirring in a vial equipped with septum. The oxygen was removed by bubbling N_2 through the solution. The polymerization was initiated by heating to 40 °C and stirred for three days at this temperature. The poly(*N*-[tris(hydroxymethyl)methyl]acrylamide) was precipitated with cold ether and dried in a vacuo until constant weight. 85 mg of the white/light yellowish solid product **5** was obtained.

78.5 mg of polymer **5** macroCTA, 0.92 mg VA-044 (0.003 mmol), and 5.90 g RAMEB (4.5 mmol) were dissolved in water and bubbled trough N_2 in a vial equipped with

Teflon covered septum. Isoprene (0.45 mL, 4.5 mmol) was added by a syringe into the vial, and the mixture was stirred until clear solution. The polymerization was initiated by heating to 40 °C and stirred for three days at this temperature. The resulting milky emulsion was heated for 20 mins at 80 °C; the white precipitate was isolated by filtration and lyophilized to obtain 260 mg of solid product.

IR = 3100-3600 cm^{-1} (O-H, N-H), 2800-3000 cm^{-1} (C-H) 1600-1700 cm^{-1} (C=O)

^{1}H NMR (DMSO d_6, 400 MHz) δ/ppm = 7.4–6.7 (br m, low intensity H-C), 5.15–4.97 (H-d), 4.95 (s, 1.2H, H-1, OH), 4.00–3.30 (m, 6H, H-2–H-6, H-B), 3.25 (s, 1.8H, O-CH_3), 2.05–1.85 (H-b, e, C) and 1.68–1.11 (H-a, D)

The total weight fraction w_{CD} = 72.0 wt % of RAMEB was determined from the optical rotation α =0.103 deg of a solution (10.65 mg mL^{-1}) in $CHCl_3$ at λ = 589 nm applying the measured specific rotation of Me-β-CD $[\alpha]_{589}$ = +130 mL $dm^{-1}g^{-1}$. For the determination of the content of free RAMEB (w''_{CD} = 8 wt %*)* a solution (total concentration of RAMEB 1.0 mM) of the polyrotaxane **6** in 0.1 M phosphate buffer was titrated at 25.000 °C with an 8 mM solution of the guest adamantane-1-carboxylate sodium salt monitored by ITC. The weight fraction of **threaded cyclodextrin** (w'_{CD}= 65 wt %) was calculated as the difference of w_{CD} = 72.0 wt % and the w''_{CD} = 8 wt % [1].

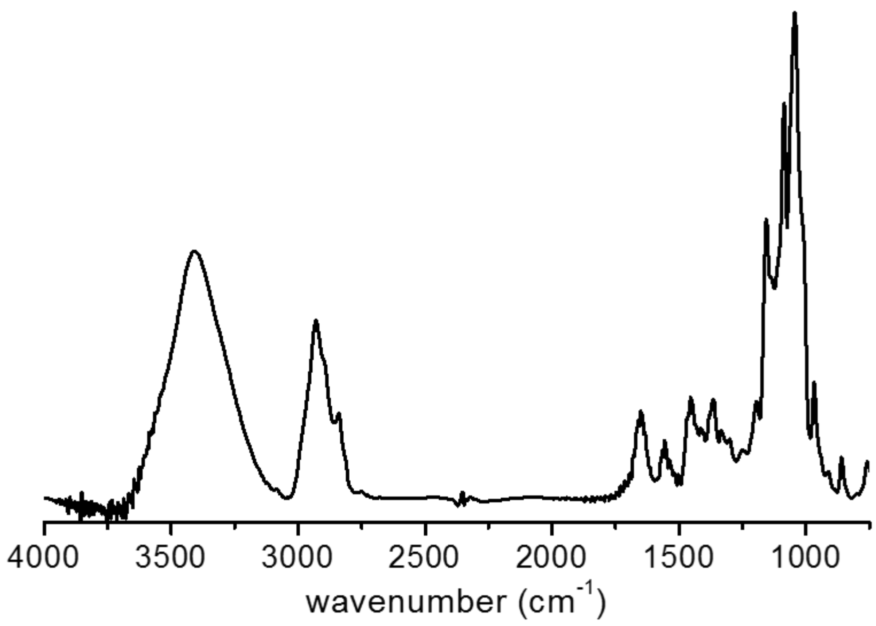

Figure S8: IR spectrum of the polyrotaxane **6**.

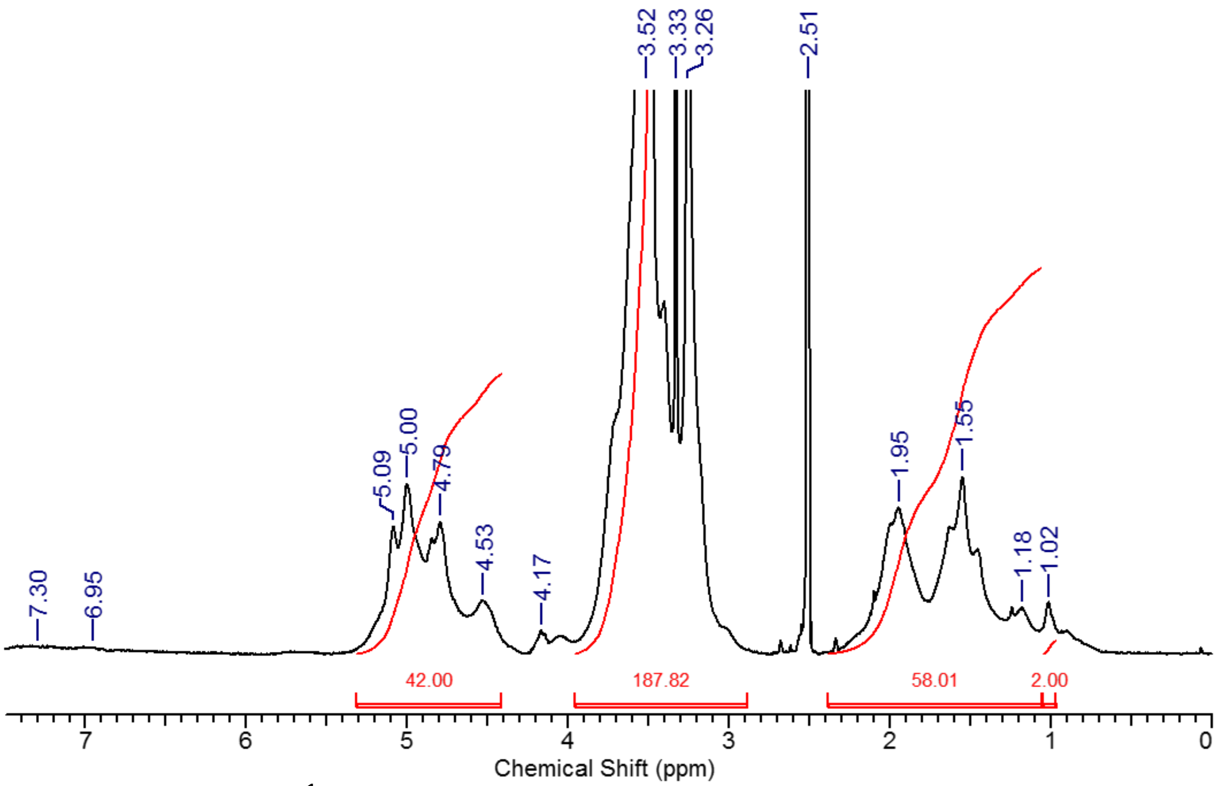

Figure S9: 400 MHz ^{1}H NMR of the polyrotaxane **6** in DMSO-d_6.

3. Determination of the molecular weight distributions

For determination of the molecular weight of the polyrotaxane by GPC, the free OH groups of 100 mg polyrotaxane **6** were acetylated by stirring it for one day in 5 mL pyridine with 0.8 mL acetic anhydride (8.30 mmol). After the reaction, the product was precipitated with cold diethyl ether, and dried under vacuum until constant weight.

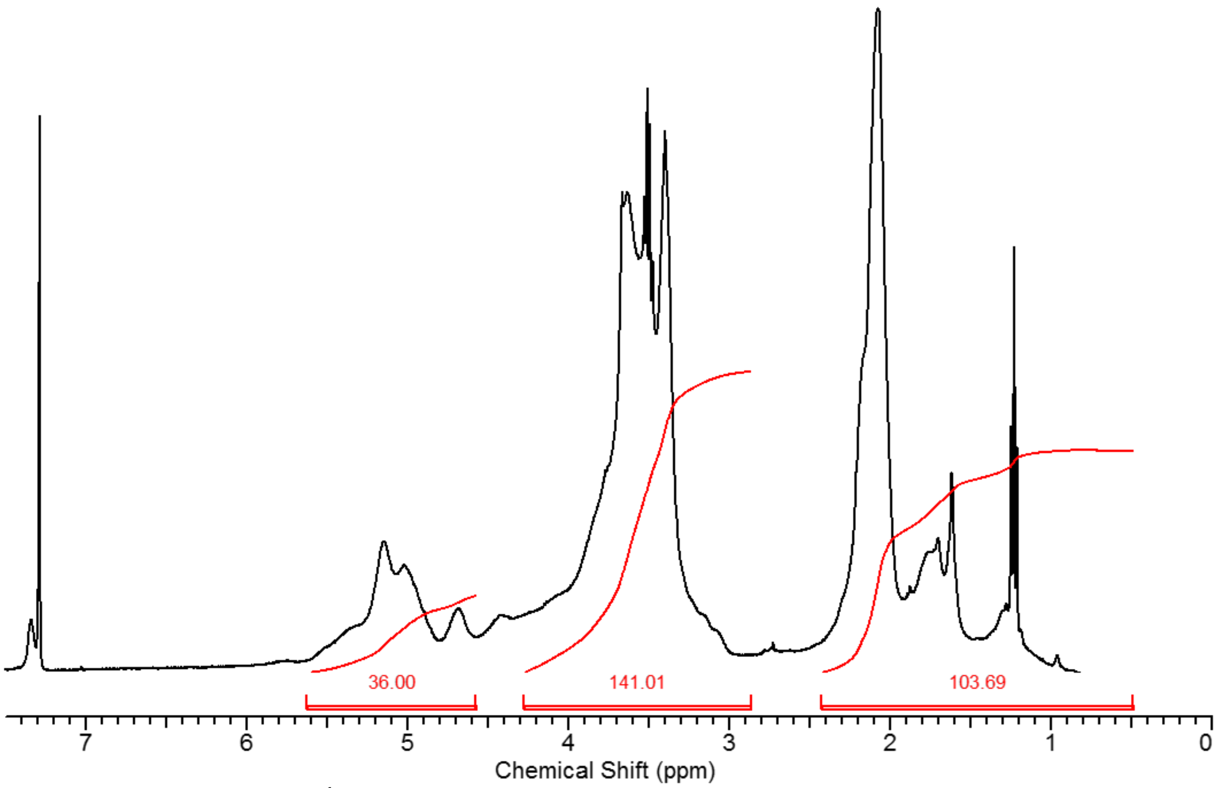

Figure S10: 400 MHz ^{1}H NMR of the acetylated polyrotaxane **1** in $CDCl_3$.

.

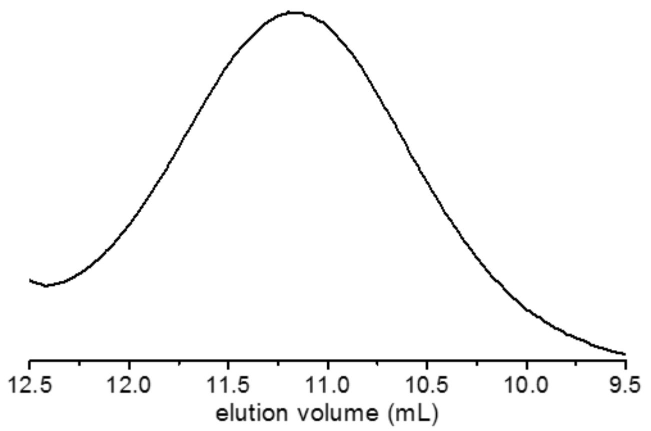

Figure S11: GPC of the acetylated polyrotaxane **2** in THF.

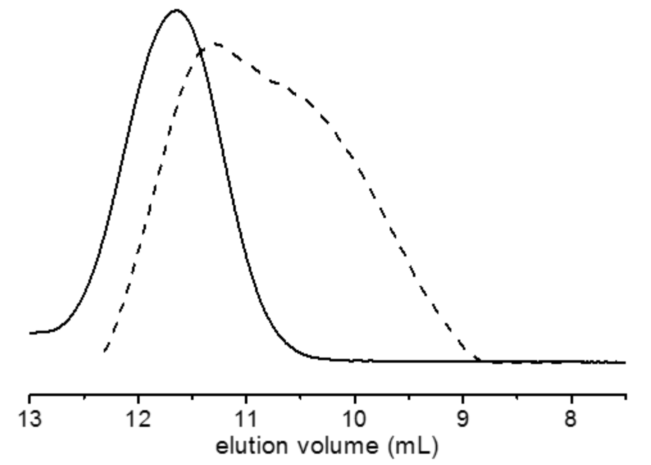

Figure S12: GPC curves of the acetylated macroCTA **3** (straight line), as well as the acetylated polyrotaxane **4** (dashed line) in THF.

References

1. Kali, G.; Eisenbarth, H.; Wenz, G. *Macromol. Rapid Commun.* **2016**, *37* (1), 67–72.
2. Lai, J. T.; Filla, D.; Shea, R. *Macromolecules* **2002**, *35* (18), 6754–6756.
